# Old Men's Children

**Published:** 1900-06-30

**Authors:** 


### OLD MEN'S CHILDREN.

Dr. Harry Campbell[1] has hit upon the happy idea that monogamy exercises an injurious influence on the race in regard to longevity. He says that the most vigorous specimens of the human male certainly retain procreative power beyond the allotted age of the Psalmist, and that we owe it to this very circumstance that many of us are able to reach or even to live beyond this limit. "Had no man during the last 100,000 years," he says, "got offspring after the age of 50 we should all probably tend to die of old age about this time. There has, however, been no such limit to reproduction. In primitive communities, *e.g.*, the man who by his great physical superiority becomes the warrior chief or king may continue to get children until well on in years, and may thus leave many more children than the average man. Now children thus got, female as well as male, tend by the law of heredity to live as long as their father, to resist deadly diseases as long as he," and he argues that such old men's children exercise a leavening influence on the race, an influence which has been at work from the time of the ape-like man, and one which in these monogamous times is checked by the fact that a man who marries one wife of about his own age loses the advantage in regard to number of offspring which would accrue to the vigorous and long-lived man under a system of polygamy, which would allow his procreative powers to operate late into life. A man "marrying a woman of about his own age, and possessing the power of propagation into advanced life, leaves no more children to inherit his superior death-resisting power than the inferior man who dies at 45. Monogamy in such a case reduces the superior man to the reproductive level of the inferior man, and robs the race of a number of individuals whose birth would diminish the racial tendency to disease in the later years, and increase the racial span of life. The effect of monogamy, in fact, is to increase the racial tendency to disease after middle life and to shorten the vital span." We are afraid that ordinary men will say that if this argument teaches anything it drifts towards Mormonism and its interesting system of rewarding success by polygamy. Dr. Harry Campbell, however, tells us that so apparently obvious a way out of the difficulty is a "sociological impossibility," in saying which he is no doubt right. He is, however, thus driven to maintain that the only way to raise the racial standard "is for the unfit to submit to voluntary elimination, and to abstain from getting unfit variations," in regard to which we may well ask: (1) Will they submit? (2) who are the unfit? and (3) are we sure that in breeding for longevity we are really breeding for the best type? Till these questions are answered to the general satisfaction of a world crowded with "unfit" specimens, we doubt whether any serious steps will be taken. The only serious attempt, in fact, that has of late years been made by any large community to deal with this question is that made by the Mormons—a step so hateful to the rest of the Anglo-Saxon race as to be a "sociological impossibility." We are not sure, however, that Dr. Harry Campbell is right in his argument. Longevity may be a mere by-product of racial stamina, and may have nothing whatever to do with the numerical superiority of the offspring of long-lived men.

[1] Lancet, June 9.

